# The Vacancy in the General Medical Council

**Published:** 1897-09-25

**Authors:** 


### THE VACANCY IN THE GENERAL MEDICAL COUNCIL.

The election by the registered practitioners of England of their "direct" representatives on the General Medical Council, although taken very seriously by a certain proportion of the electors, has not been by any means devoid of some of the elements of comedy; and the curtain has just been rung down upon one act of the drama, in which these elements have been unusually abundant. The last election, it will be remembered, was held in December, 1896, on which occasion two of the former direct representatives, Sir Walter Foster, M.P., and Mr. Wheelhouse, declined to be put in nomination, on the ground, as it was generally stated and believed, of the claims made upon them by certain "reformers" demanding that they should attempt undertakings which were wholly outside the province of the Council, and beyond the limits of its statutory powers. Ten candidates, many of whom were liberal in their promises to effect impossibilities, came forward, and 22,577 voting papers were sent out. About half of the profession abstained from voting, but 11,990 papers were correctly filled up, and 6,646 votes were recorded for Dr. Robert Reid Rentoul, of Liverpool, who was thus returned at the head of the poll, with Mr. Brown and Dr. Glover as his colleagues. Dr. Rentoul was chiefly known by the pains which he had taken to put forward his views on the subject of projected legislation to provide for the proper education and the registration of midwives, a subject with which the Medical Council appears to have had no further concern than such as arose from its having more than once been asked to advise the Government with regard to it. In ordinary circumstances there would have been no opportunity of bringing any reference to it within the Council agenda, but Dr. Rentoul was so far favoured by fortune that in the May session, when he first took his seat as a representative, the opinion of the Council was asked by the Privy Council on the provisions of a Bill for the Registration of Midwives, which was then before Parliament. It was known that the Bill could not be carried this year, but that it would probably be brought forward again; and, in these circumstances, and in order to afford ample time for the discussion of an important question, the Council decided to let the matter stand over until the November session. In the meanwhile a committee, of which Dr. Rentoul was a member, was appointed to report on the Bill, and to prepare the way for any action which the Council might be disposed to undertake.

Dr. Rentoul, like his colleagues, had been elected for a term of five years, and he had no doubt been elected specially on account of his expressed willingness to protect the interests of his brethren with reference to the midwifery question. The Bill was delivered into his hands, and he possessed a golden opportunity, of which, we think, he was bound to avail himself considering the position he had taken up before his election. But on September 1st Dr. Rentoul wrote to the President of the Council a letter containing only these words, "Please accept my resignation of membership of the General Medical Council." The President had no choice but to do as he was requested. He acknowledged the letter, officially notified the vacancy, and summoned a meeting of the English Branch Council for the 28th instant, to receive the nominations of candidates and to give directions for taking the poll. Several nomination papers had been asked for and supplied, when, last Saturday, Dr. Rentoul sent a telegram to the President, announcing that he withdrew his resignation, and also sent telegrams to the same effect to a number of newspapers. The position has been laid before the legal advisers of the Council for their opinion; but, in the meanwhile, the common-sense view of the matter is that the resignation is complete, that Dr. Rentoul ceased on September 1st to be a member of the Council, and that nothing which he has done since can restore him to that position. The candidates already in the field have a clear right to the vacancy, and would have a distinct grievance if it were declared not to exist.

